# Whole Genome Duplications and a ‘Function’ for Junk DNA? Facts and Hypotheses

**DOI:** 10.1371/journal.pone.0008201

**Published:** 2009-12-14

**Authors:** Reiner A. Veitia, Samuel Bottani

**Affiliations:** 1 CNRS-UMR 7592, Institut Jacques Monod, Paris, France; 2 CNRS-UMR 7057, Laboratoire Matières et Systèmes Complexes (MSC), Paris, France; 3 Université Paris Diderot, Paris 7, Paris, France; Purdue University, United States of America

## Abstract

**Background:**

The lack of correlation between genome size and organismal complexity is understood in terms of the massive presence of repetitive and non-coding DNA. This non-coding subgenome has long been called “junk” DNA. However, it might have important functions. Generation of junk DNA depends on proliferation of selfish DNA elements and on local or global DNA duplication followed by genic non-fonctionalization.

**Methodology/Principal Findings:**

Evidence from genomic analyses and experimental data indicates that Whole Genome Duplications (WGD) are often followed by a return to the diploid state, through DNA deletions and intra/interchromosomal rearrangements. We use simple theoretical models and simulations to explore how a WGD accompanied by sequence deletions might affect the dosage balance often required among several gene products involved in regulatory processes. We find that potential genomic deletions leading to changes in nuclear and cell volume might potentially perturb gene dosage balance.

**Conclusions/Significance:**

The potentially negative impact of DNA deletions can be buffered if deleted genic DNA is, at least temporarily, replaced by repetitive DNA so that the nuclear/cell volume remains compatible with normal living. Thus, we speculate that retention of non-functionalized non-coding DNA, and replacement of deleted DNA through proliferation of selfish elements, might help avoid dosage imbalances in cycles of polyploidization and diploidization, which are particularly frequent in plants.

## Introduction

C-value is defined as the haploid DNA content of an organism [1]. The lack of correlation between genome size and organismal complexity, the “C-value paradox”, is accounted for by polyploidy and the expansion of repetitive DNA [2]. Repeats and non-coding, apparently nonfunctional, DNA are what Ohno called “junk DNA” [3]. Much attention has been devoted to this part of the genome, especially since 1980, when the term “selfish” DNA was introduced to designate sequences that propagate themselves within a genome, without contributing to the development of the organism [4], [5]. The selfish DNA hypothesis is selectionistic at the gene level but rather neutralistic from the perspective of the organism and the population. However, numerous works have proposed potential functions and phenotypic effects for non-coding DNA. Transposable elements are the main source of repetitive DNA and can affect gene structure and expression in several ways by promoting genomic rearrangements [6]. An analysis of repetitive elements in two insects led to the idea that these sequences might be considered as genomic symbionts under cellular regulation. Indeed, von Sternberg et al. (1992) proposed that these elements may have originated as selfish sequences and subsequently acquired functions as a result of a coevolution with other, often physically close, DNA segments [7]. Moreover, repetitive elements can interfere with transcription control or even become part of open reading frames [8]. In plants, during polyploidization events, retroposon activation may drive the synthesis of antisense or sense transcripts from adjacent sequences involving known genes. This phenomenon is associated with silencing or overexpression of the corresponding genes, respectively [9]. The abundance of transposable elements in genomes and their ability to be activated by various signals supports the view of transposons as potential controlling elements, adaptative or not [9]. Interspersed elements are also important components of animal genomes. Interestingly, about 20% of eutherian conserved non-coding sequences (CNS) involved in gene regulation are recent inventions postdating the divergence with marsupials and come from sequences inserted by transposable elements [10], [11]. Transposons have also been the source of important proteins for vertebrates, such as the site-specific recombinases Rag1 and 2 ([12] and references therein).

Other authors have proposed global adaptive roles for junk DNA as scavengers of intranuclear chemical mutagens ([13] and references therein), because an excess of non-coding over coding sequences would decrease the probability of mutations in the latter. Indeed, the number of nucleotides damaged by mutagens in coding sequences is expected to be inversely proportional to the size of the non-coding DNA fraction.

Genomic DNA content is positively correlated with nuclear and cell volumes in a wide range of organisms [14], [15]. Indeed, bulk DNA, independently of its sequence, seems to determine cell volume as a result of a “nucleotypic effect” [16], [17]. Along similar lines, the nucleoskeletal hypothesis posits that optimal cellular function would require a rather constant nucleo/cytoplasmic (karyoplasmic) ratio ensuring an optimal exchange between the two cellular compartments. This implies that DNA itself or its associated proteins should play an architectural role in maintaining nuclear volume, which would in turn dictate cell volume ([18] and references therein). The most striking example of the relationship between genome size and cell volume is provided by ploidy series (i.e. nuclear and cell volumes increase with ploidy level) [2]. This has been clearly shown for yeast autopolyploids (see [19]). Interestingly, and relevant to what is discussed below, an increase of the nuclear volume also decreases the flow of mutagens, coming through its surface, per unit of nuclear volume ([20] and references therein).

In this theoretical paper we speculate that proliferation of selfish DNA and by extension the retention of seemingly nonfunctional DNA can have other ‘functions’ connected with the physical properties of the cell which might be critical to ensure the balance between interacting gene products after whole genome duplication (WGD) events.

## Results and Discussion

### Replacing Superfluous Coding DNA by Non-Coding DNA in Polyploids: Avoiding Dosage Imbalances

**Figure 1:**
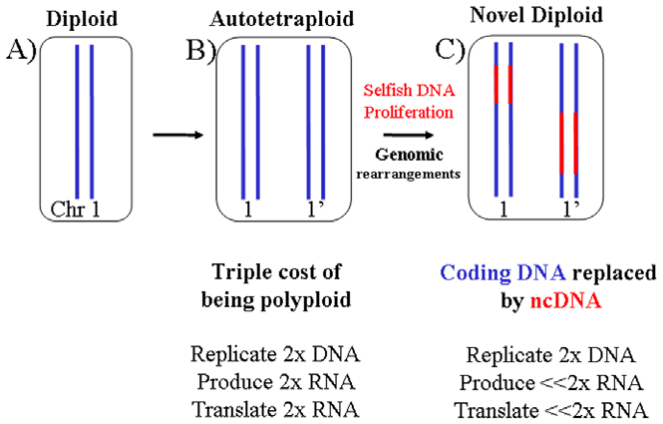
The triple cost of polyploidy. A) Original diploid cell. Chromosomes are represented as blue lines. B) Cell after whole genome duplication (WGD). Notice that the cellular volume has doubled. C) After WGD superfluous gene copies can become junk DNA or be replaced by selfish DNA. This avoids paying the cost of transcription and translation of vast genomic regions and contributes to the rediploidization process.

There is increasing evidence supporting the idea that some stoichiometric balance between and within the subunits of macromolecular complexes must be maintained to ensure their normal functioning [21]–[24]. Dosage balance should also be maintained in cellular circuits and networks where there are opposing forces such as a kinase versus a phosphatase or a transcription activator versus and inhibitor [25]. After polypolidization, duplicated genes encoding interacting proteins that are dosage sensitive tend to survive together because deletion of one copy would mimic an aneuploid effect. Regulatory genes that are in balance can be preserved from non-functionalization for millions of years and this has been observed in *Arabidopsis*, rice and other organisms [24] and references therein). On the other hand, genomic analyses and experiments have provided evidence that after a WGD there is a strong tendency to go back to a diploid state, suggesting that diploidy is the most stable state [26]. Indeed, DNA deletions due to intra/interchromosomal rearrangements and chromosome losses owing to segregation defects [27] are concomitant with (and facilitate) the return to a diploid state. Deletion of genes that are not necessary in multiple copies can be advantageous because their expression imposes a triple cost to the cell: futile replication, transcription and translation (Figure 1). However, such deletions might indirectly affect gene-product dosage balance and, as discussed below, in most cases they should not be massive and rapid.

By virtue of the nucleotypic effect of DNA, DNA deletions in a newly formed polyploid is expected to decrease nuclear and cell volumes. Assuming that such a hypothetical volumetric contraction does not alter substantially transcription levels on a per-allele basis, it would lead to an increase in the concentration of the products of genes that remain as duplicates. This might be advantageous for a subset of genes (as has been previously proposed [28]) but not for all. Here, we explore the idea that proliferation of non-coding DNA compensates for DNA deletion after a WGD and helps stabilize the nuclear/cell volume, thereby preserving the balance between gene product concentrations. We will illustrate this point with several examples.

**Figure 2:**
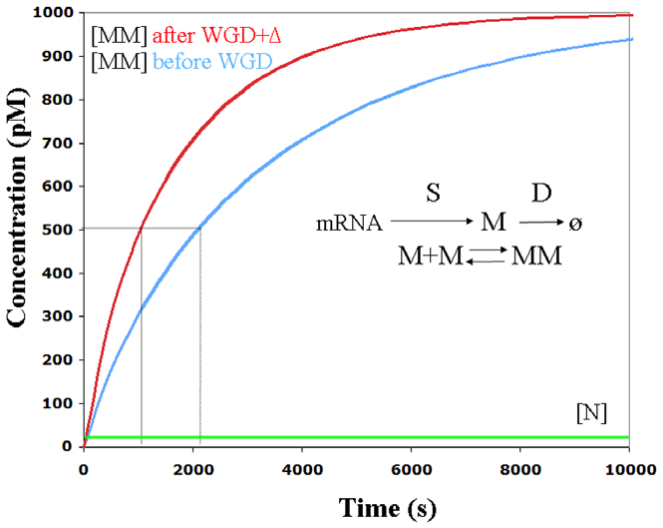
Dynamics of the formation of the dimer MM (in balance with monomer N) and genome duplication. Formation of MM depends on the synthesis rate S, the degradation of the coding mRNA and monomers (D) and the interaction of the monomers. Blue curve: dimer formation after WGD (parameters S = D = 1) and red curve dimerization after WGD+deletions (leaving only M, N and the protease-encoding genes as duplicates, S = D = 2). Notice that the steady state is reached more rapidly in the latter system (red curve) than in the orginal tetraploid or diploid (blue curve). Such a kinetic difference can be crucial, especially especially if time delays (as in the mitotic clock of figure 3) are important. If MM is in balance with monomers N, there might be a problem before reaching the steady state.

Let us first consider the case of the dimer, MM, in balance with a monomer, N. For example, MM and N might be enzymes or transcription factors with opposing activity. As shown in figure 2, the process of formation of MM is a function of the rate of synthesis (S) of M, its proteolytic degradation rate (D) and dimerization itself (more details in the Materials and Methods sections). After autotetraploidization, the right balance is maintained because expression of both M and N is increased with ploidy along with the volumetric increase. Of course, if one paralogous copy of the genes encoding either M, N or the protease is deleted, an imbalance will appear. Thus, it is likely that during non-functionalization and DNA deletion that follows a WGD, the trio of M, N and the protease-encoding genes will tend to be retained. Let us now explore what would happen after a hypothetical (and extreme) volumetric contraction due to a ‘massive” DNA deletion (scenario ‘WGD+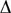
’). Under this assumption, if M is expressed in response to a signal, the kinetics of formation of MM before reaching the steady state is altered as compared to the initial autotetraploid state, even if all interacting genes are retained. Figure 2 shows the kinetics of an extreme situation where all duplicates have been deleted but those involved in the system MM-N (i.e. WGD+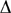), compared to the initial one. Both systems attain the same concentration of MM at the steady state (as predicted in [25]). However, this process is faster in the case of ‘WGD+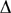
’. Indeed, to attain the halfpoint of the steady-state concentration of MM, the initial cell requires twice as much time as the one in the situation WGD+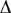. In turn, N, which acts as a monomer, attains the steady state much more rapidly in both systems. Such a kinetic difference predicted for MM can be crucial, especially in signal transduction cascades and other cellular pathways where the kinetics, and time delays, are important. This holds also for cases involving a slow/progressive deletion process.

**Figure 3:**
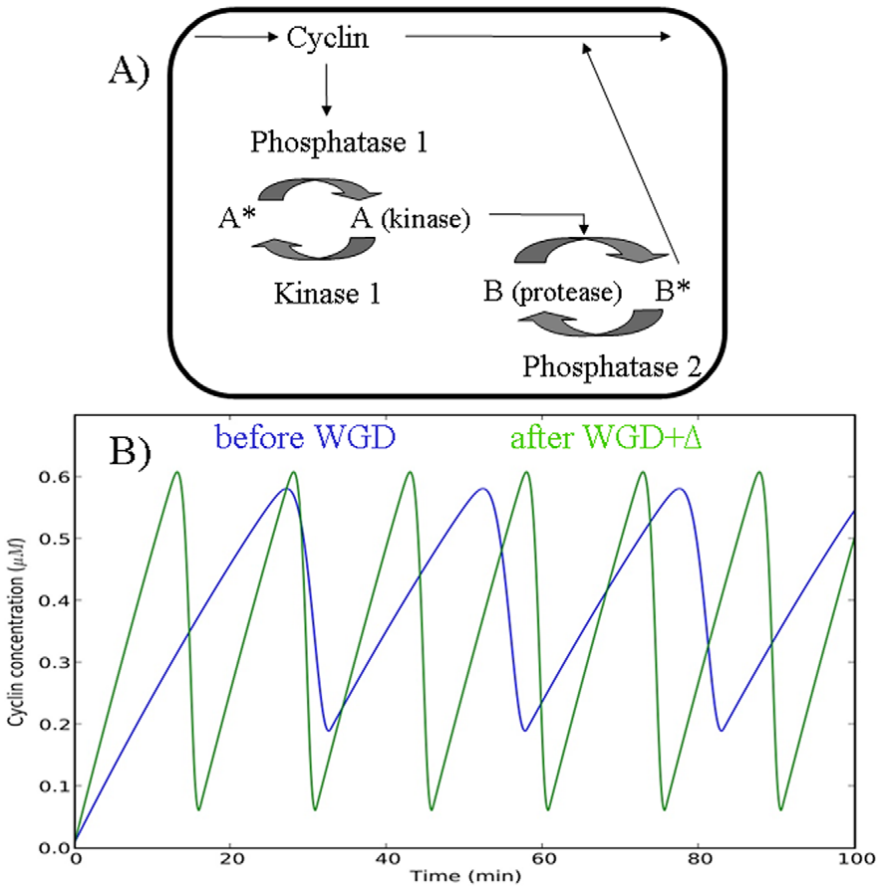
Kinetics effects of WGD and deletions. A) Outline of a minimal mitotic cell cycle model [29], based on a cascade of post-translational modifications that modulates in the end a protease degrading a cyclin. Such a negative feedback loop generates oscillations. B) The blue curve is the periodic variation of cyclin with the set of parameters of Goldbeter (1991). The green curve (with faster cycling) corresponds to parameters for doubled enzyme concentrations resulting from a WGD followed by extensive DNA deletions.

Increases of the concentrations of gene products involved in cellular circuits (i.e. in an evolutionary time-scale due to deletions+volumetric contraction) can also upset the regulation of the latter and change their dynamics. To explore such effects we turn to the minimalist model of a mitotic clock [29]), which reproduces qualitatively some features of the cell cycle. Again, we will consider an extreme scenario in which, after a WGD and subsequent DNA deletion, only genes involved in the model circuit are left duplicated (WGD+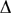). This doubles the concentrations of the corresponding gene products (and of their synthesis and degradation rates). Figure 3 shows that these conditions drive the circuit dynamics to a potentially unsustainable regime, namely to doubling the frequency of the cell-cycle.

The examples above show that even if dosage balance is maintained *stricto sensu*, a potential volumetric contraction owing to DNA deletions might be harmful. Thus, some of the existent non-coding DNA (including repeats) may have a connection with maintaining optimal regulation of gene expression after a WGD, as previously proposed in a different context [30]. Transformation of coding regions into non expressed (non-transcribed/non-translated) pseudogenes and allowing selfish DNA proliferation (i.e. replacing deleted DNA) might help stabilize the nuclear/cellular volume and thus, the functioning of cellular circuits and pathways. According to this scenario, non-functionalized genes and selfish DNA are obviously not completely devoid of function.

**Figure 4:**
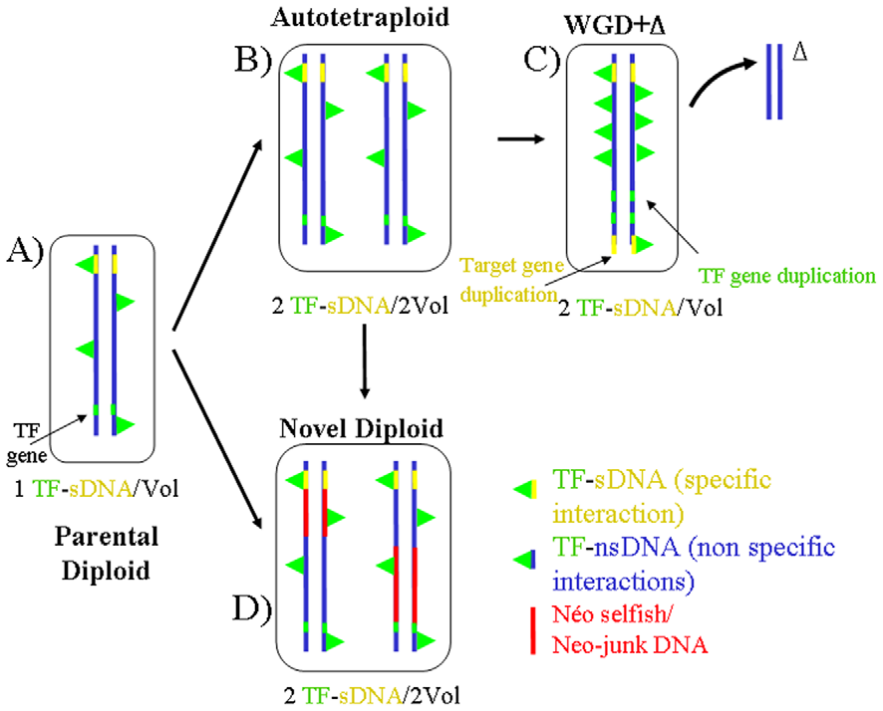
Non-specific protein-DNA interactions and WGD. A) Original diploid cell. Blue lines: chromosomes, green segments on the chromosomes: TF-encoding gene, yellow chromosomal segments: specific TF target binding sites, green triangles: TF protein. B) Cell after WGD. The cell volume has doubled and the concentrations of bound sites in the tetraploid (specifically or non-specifically) are the same as in the original cell. C) Cell after WGD+DNA deletions. Duplicated ‘superfluous’ DNA is removed leading to a volume shrinkage. This leads to doubling the concentration of TF-sDNA (specific interactions) with respect to the original autopolyploid or tetraploid. D) WGD+generation of junk/selfish DNA that replaces deleted DNA (red lines). Duplicated chromosomes are differentiated (diploidization) and cell volume is similar to that of the original tetraploid and the concentrations TF-sDNA and TF-nsDNA are respected.

**Figure 5:**
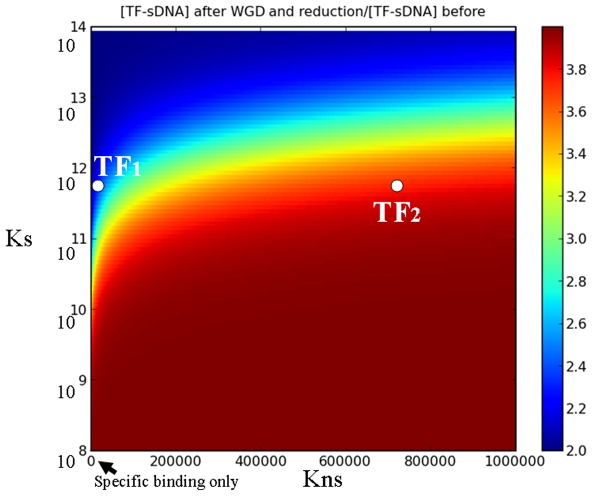
Quantitative exploration of specific transcription factor binding in the presence of different levels of non specific binding. Quantitative exploration of specific transcription factor binding in the presence of different levels of non specific binding. Consider a TF ([TF] = 1 nM) that specifically recognizes 10 binding sites/nucleus. Specific recognition takes place with Ks' ranging between 10^8^ to 10^14^) while non-specific recognition takes place with much lower affinity. Intranuclear concentration of specific target sites is about 3.10^−11^M (assuming a nuclear volume of 5.10^−13^L). The initial concentration of irrelevant DNA binding sites is assumed to be 7 orders of magnitude higher than sDNA. The color scale represents the ratio of the concentration of TF bound to specific site on DNA in the case WGD+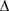 (leaving TF and its targets duplicated) over the concentration of TF bound to specific sites before WGD. For low non-specific binding the concentration of specifically bound TF targets in WGD+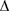 is twice as much as in the case without WGD (blue zone). In presence of significant non-specific binding, the concentration of specifically bound sites can be as much as 4× higher than without duplication as the synthesis of TFs is doubled whereas non-specific binding sites available for sequestration are in identical concentration. The example of TF1 and TF2 (in balance) is displayed. TF1 and TF2 have the same global concentration but TF1 binds only specifically and TF2 has substantial non-specific binding. Under the scenario WGD+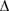, TF2 might form as much as two times more complexes than TF1, which obviously would perturb their balance.

Another outstanding biophysical effect of non-coding DNA that cannot be overlooked in a WGD process involves protein-DNA interactions (and by extension, protein-membrane interactions). DNA binding proteins may recognize sequences that are similar to their real target sites giving rise to non-specific interactions ([31] and references therein). This is obvious for proteins such as basic-HLH and leucine zipper-containing factors that have a basic DNA-binding domain, allowing non-specific electrostatic interactions with DNA. Given the size of eukaryotic genomes, the amount of DNA available for non-specific interactions is enormous with respect to the specific binding sites for a particular factor. For simplicity, we disregard potential differences in the contribution of euchromatin and heterochromatin to non-specific binding. The existence of a substantial amount of non-specific interactions is likely to pose a problem when genomic DNA is deleted and not replaced. This can be explored by the analysis of the binding of a transcription factor, TF, to specific (sDNA) and non-specific (nsDNA) sites. In the context of a recently formed tetraploid, let us consider a TF that specifically recognizes a few binding sites/nucleus. Specific recognition will take place with high affinity (affinity constant Ks) while non-specific recognition will normally take place with much lower affinity (Kns). The concentration of irrelevant DNA binding sites can be several orders of magnitude higher, which can easily be the case in plant genomes, because each short sequence is in principle a non-specific binding site. Now let us focus on an extreme case (as in the examples above) where after a WGD there is deletion of all extra non-coding DNA and only the genes encoding the TF and its targets are left as duplicates (Figure 4). By virtue of the nucleotypic effect, the nucleus should undergo a 2-fold volumetric shrinkage, which translates into doubling the concentration of the TF and its targets while the concentration of nsDNA will remain approximately the same (i.e. half DNA amount, as compared to the tetraploid, in half the volume). If there were only specific binding, coming back to the ancient volume while retaining double doses of TF and its target sequences implies doubling the concentration of the complexes TF-sDNA. On the other hand, in presence of non-specific binding, the same amount of TF is normally shared by sDNA and nsDNA sites and the results are quite different: the higher the non-specific affinity Kns, the higher the concentration of complexes TF-sDNA formed after a hypothetical genomic shrinkage. In other words, a double amount of TF produced after genomic shrinking, for a smaller concentration of non-specific binding sites, leads to a non-linear increase in the effective TF concentration and thus in the concentration of TF-sDNA complexes. These changes in the binding of TFs to their specific targets can alter the behavior of genetic networks significantly. Consider for instance what would happen to a network involving two different factors, TF1 and TF2 that are in balance. We will assume, for simplicity, that in the steady state they both reach the same global concentration and have the same Ks. If they do not undergo non-specific binding, there will be no problem (i.e. both TF1-sDNA and TF2-sDNA concentrations are doubled after WGD+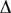). However, if for instance TF1 binds only specifically but TF2 has substantial non-specific binding, TF2 can form as much as two times more complexes than TF1, which should perturb their balance (Figure 5). Again, a strategy that keeps non-specific interactions at optimal levels involves i) pseudogenization without deletion or ii) replacement of deleted DNA by repetitive DNA.

### General Discussion and Conclusions

The evolution of C-value in polyploids is influenced by i) the deletion of structural genes (as their transcription and translation is costly), ii) the retention of structural genes whose products are required at high doses and of balanced regulatory genes that enhance the generation of evolutionary innovation and plasticity [24], [32] and iii) the multiplication of interspersed repeats. As proposed above, the potentially negative impact of deletions after a WGD can be buffered if deleted genic DNA is, at least temporarily, replaced by repetitive DNA in such a way that the nuclear/cell volume remains compatible with normal living. Later, the equilibrium between deletions and proliferation of non-coding DNA can be biased towards a new point involving changes in C-value. This assumption is required to explain the wide spectrum of DNA contents observed even within a plant species.

Selfish DNA proliferation has been observed during polyploidization events. For instance, the steady-state transcript levels of some retrotransposons are much higher in newly synthesized wheat amphiploids [9]. Bursts of transposon activity have been described in other cases, as in *Oryza australiensis* [33]. Several DNA transposons in newly synthesized *Arabidopsis* allopolyploids, also display transcriptional activity, although their transposition is limited [34]. A burst of expansion has also been linked to the repeated formation of active recombinant elements derived from two parental retrotransposons brought together during allopolyploid formation [35].

Speciation by allopolyploidization involves complex interactions between the merging genomes. After allopolyploidization, most genes tend to be expressed at mid-parental levels but for a proportion of them, the transcriptional contributions of each subgenome are not additive, that is, each sub-genome dominates with regard to the expression of a set of genes [36]. However, it is conceivable that, when allopolyploidization involves genomes of very different C-values, a sudden change in the extent of non-specific TF-DNA interactions might lead to a global dominance of one parental subgenome over the other. Intuitively, it is expected that expression from the large parental genome would be favored because “concentrations” of cis-regulatory elements are lower in the nucleus of large genomes and competition by non-specific binding targets is greater. Thus, this may demand the evolution of higher affinity (and/or more concentrated) TFs and more efficient cis-regulatory elements. A test for this prediction would require the merged genomes to be as evolutionarily close as possible to control for the contribution of the molecular divergence of the merged networks to non-additive gene expression.

All in all, we propose that at some point in the evolution of polyploids, junk DNA, including selfish elements, may have played (or play) an adaptive role linked to global functional effects of DNA. We hope that this theoretical exploration will provide some insights into the process of genome evolution.

## Materials and Methods

### Simulations

The differential equations models for the kinetics of multimer formation before and after WGD+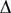 (figure 2) and for the mitotic cell cycle before and after WGD+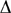 (figure 3) were simulated with the xpp/xppaut integration program (B. Ermentrout, http://www.math.pitt.edu/~bard/xpp/xpp.html). The concentration profile of TFs bound specifically to target sites as a function of different levels of non specific binding and specific binding strengths (figure 5) was plotted with the python matplotlib package.

### Simple Model of Dimer Formation

We consider a simple model of formation of a protein dimer MM: the monomer M is synthetised from the gene and mRNA with rate 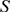; the monomers associate with rate 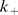 to form the dimer MM, that dissociates with rate 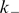; the monomers degrade with rate 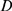:
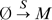
(1a)

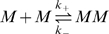
(1b)

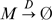
(1c)described by the kinetic equations:
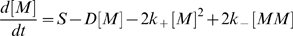
(2a)

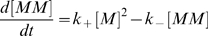
(2b)


To study the effect of Whole Genome Duplication followed by DNA deletions (WGD +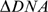), we consider the limit case where all genes are duplicated, then the rates are doubled as twice the monomer mRNA amount is produced (in the limit case we assume the same recovered initial volume is recovered after massive DNA reduction), and also twice the amount of enzymes in the degradation chain: 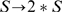, 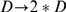. On another hand the association and dissociation rates 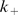 and 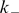 remain unaffected by changes in gene dosage.

The steady state concentrations 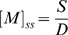 and 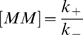 are the same before and after WGD+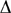, however integration of the differential equation system above gives different kinetics when synthesis and degradation rates are doubled as shown in figure 2.

### Minimal Mitotic Cycle Model

The minimal mitotic cell cycle model of Goldbeter (1991), figure 3, is described by the following system of kinetic equations:
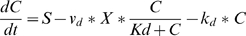
(3a)

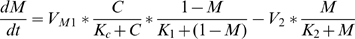
(3b)

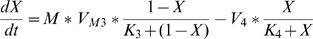
(3c)


In the above equations, 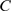 denotes the cyclin concentration and 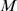 and 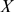 are the fraction of respectively active cdc2 kinase and of the active cyclin protease. The parameters 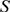 and 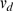 denote respectively the maximum rate of cyclin synthesis and the maximum rate of cyclin degradation; 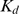 and 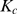 denote the Michaelis constants for cyclin degradation and for cyclin activation of the phosphatase; 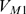, 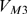, 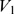, 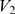 are the maximal rate of the relevant enzymes either for phosphorylation and dephosphorylation. The parameters 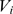 and 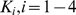 are normalized by the total amount of the relevant enzyme.

Assuming doubling of the concentrations of *all* the genes involved after a Whole Genome Duplication followed by DNA reduction (WGD+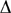) and volume shrinkage, the parameters change in the following way:

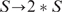
, the cyclin synthesis rate is proportional to the amount of cyclin mRNA.

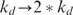
 the first order degradation rate doubles if the concentration of all genes in mRNA degradation pathways are doubled, this parameter has in any case not much influence as it is much smaller than 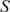.

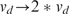
 as 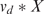 is the maximal degradation velocity proportional to the protease X concentration; as 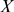 is a molar fraction if the protease amount is doubled, the maximal enzymatic velocity 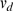 is doubled.

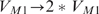
, 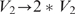 as the maximal activation and degradation velocities for the Cdc2 kinase are proportional to the amounts of respectively a phosphatase and a kinase, whose amounts double in the case of WGD+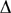.

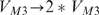
, 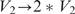 as the maximal activation and degradation velocities for the cyclin protease X are proportional to the amounts of respectively a kinase and a phosphatase, whose amounts double in the case of WGD+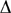 DNA.

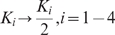
 due to the normalisation of these Michaelis-Menten by the total amounts of their related enzymes (kinase Cdc2 and protease X); doubling these concentrations leads to cutting by half these constants.

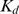
 and 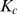 are non normalized Michaelis-Menten constant independent from gene concentrations.

The simulations of figure 3 compare the kinetics of the previous model before WGD+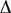 and after, with the changes of paramter as discussed.

### Transcription Factors Specific Binding and Whole Genome Duplication+DNA Reduction

To calculate the effect of non specific protein-DNA binding on transcription factor activity after whole genome duplication and massive DNA deletion let us consider a transcription factor protein 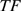 that binds specifically target sites 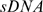 on the DNA with dissociation constant 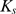 and also binds DNA non-specifically at binding sites 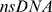 with dissociation constant 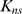 (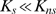):
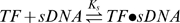
(4a)

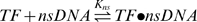
(4b)


At equilibirum we have:
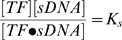
(5a)

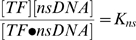
(5b)since 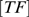 is the free TF concentration, by conservation we have also:
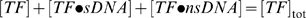
(6)


Using these relations it is simple to express the concentration of specific bound TF-DNA complexes 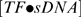 as a function of the whole TF contentration 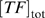:
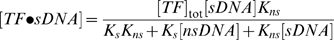
(7)


We consider now a whole genome duplication event followed by massive DNA deletions that are accompained by volumetric shrinkage. For the point of illustrating the effect of unbalance between specific and non specific binding under such events we suppose here the limit case where the the gene and specific promoter sites concentrations double, while DNA deletion leads to the same amount of non-coding DNA available for non specific biding:
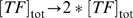


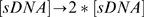


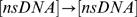
With these parameter changes, we obtain immediately the concentration of specifically bound transcription factors after WGD+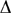:
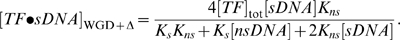
(8)


Figure 5 presents the ratio 
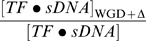
 of the specifically bound transcription factors concentration after WGD+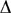 over the concentration before this evolutionary event.
